# Knife-assisted incision for restoring esophageal lumen after surgical exclusion

**DOI:** 10.1055/a-2239-3237

**Published:** 2024-03-14

**Authors:** Francesco Azzolini, Ernesto Fasulo, Francesco Vito Mandarino, Alberto Barchi, Silvio Danese

**Affiliations:** 1 9372 Department of Gastroenterology and Gastrointestinal Endoscopy, IRCCS Ospedale San Raffaele, Milan, Italy; 2 18985 Vita-Salute San Raffaele University, Milan, Italy

Surgical repair with esophageal exclusion is a life-saving surgery for patients with mediastinitis following mid-esophageal perforation 1 . This is followed by either spontaneous recanalization of the organ or subsequent surgery to restore lumen patency 2 .

We present the case of a patient who underwent endoscopic restoration of the esophageal lumen after unsuccessful spontaneous recanalization following esophageal exclusion.

A 41-year-old man, with known achalasia, underwent pneumatic endoscopic dilation at another center, resulting in a 6 cm longitudinal laceration of the lateral esophageal wall. The patient developed mediastinitis and was treated by surgical repair of the laceration and esophageal exclusion with proximal staple line division.

At 4 months post-surgery, the patient continued to experience dysphagia with a liquid diet. Postoperative esophagograms revealed poor contrast passage across the staple lines. The patient was referred to our unit for endoscopic recanalization ( Video 1 ). Endoscopically, we found a moderate stenosis (caliber 6 mm) at the staple line site ( Fig. 1 ).

Endoscopic esophageal lumen recanalization after surgical exclusion.Video 1

**Fig. 1:**
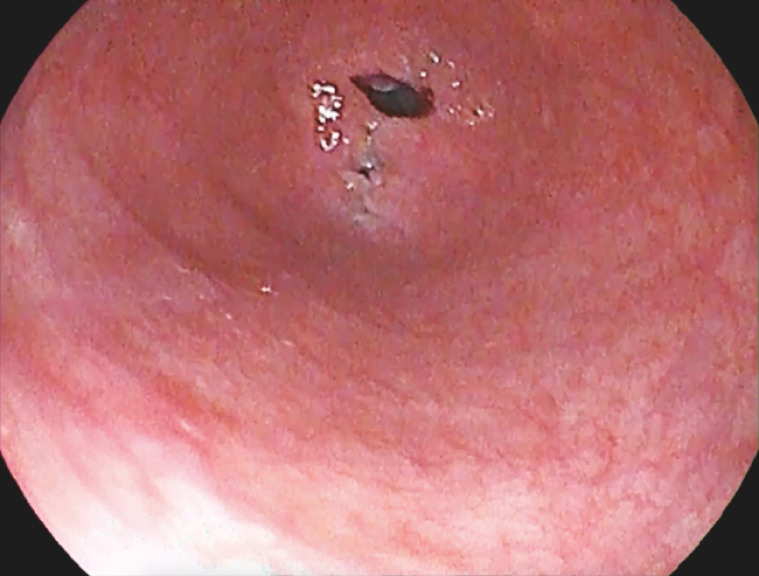
Initial appearance of the esophageal lumen.

Initially, we placed a guidewire in the stapled lumen and performed dilation with Savary–Gilliard bougies up to 9 mm. Then, we extensively incised the fibrosis between the residual lumen and the stapled lumen using an L-type dissector (Finemedix, Daegu, Korea) ( Fig. 2 ). Finally protruding staple sutures were removed by cold forceps.

**Fig. 2:**
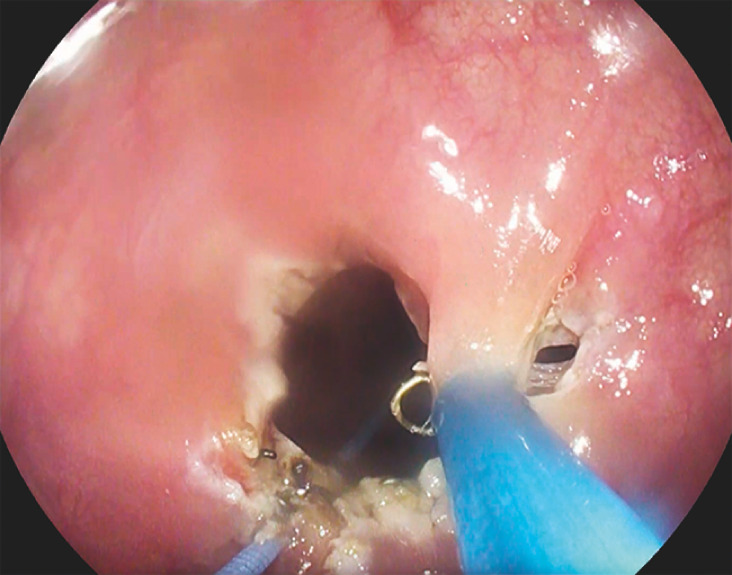
Incision of the fibrosis with L-type dissector (Finemedix, Daegu, Korea) to separate staples.

As a result, a well-patent esophageal lumen, traversable with a standard gastroscope (caliber 9.8 mm), was achieved ( Fig. 3 ). No leaks were detected on the intraprocedural esophagogram.

**Fig. 3:**
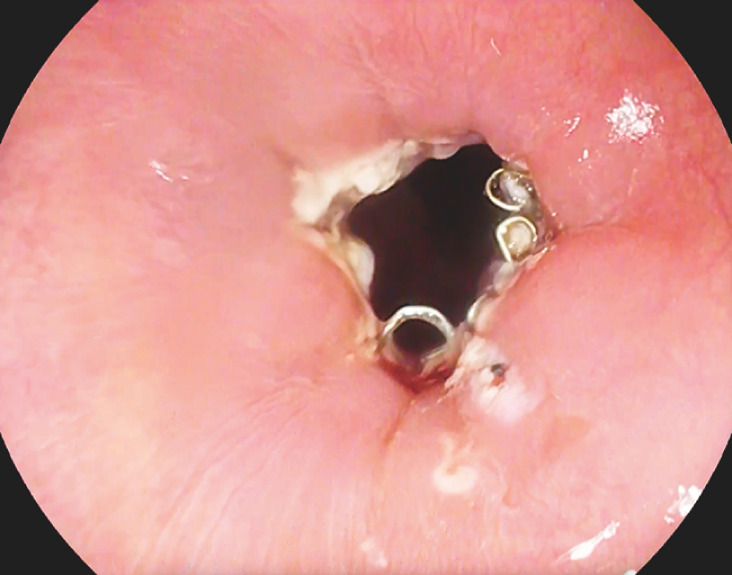
Final view: the staple line site was traversed by a standard gastroscope.

On the first postoperative day, an X-ray with contrast medium showed smooth contrast passage throughout the esophagus. The patient was discharged after resuming a soft diet. At the 3-month follow-up, he reported having no dysphagia.

To the best of our knowledge, this is the first report of endoscopic recanalization after surgical esophageal exclusion and describes a potential treatment option for similar complex cases.

Endoscopy_UCTN_Code_TTT_1AO_2AH

## Notes

Endoscopy E-Videos https://eref.thieme.de/e-videos
*E-Videos* is an open access online section of the journal *Endoscopy* , reporting on interesting cases and new techniques in gastroenterological endoscopy. All papers include a high-quality video and are published with a Creative Commons CC-BY license. Endoscopy E-Videos qualify for HINARI discounts and waivers and eligibility is automatically checked during the submission process. We grant 100% waivers to articles whose corresponding authors are based in Group A countries and 50% waivers to those who are based in Group B countries as classified by Research4Life (see: https://www.research4life.org/access/eligibility/ ). This section has its own submission website at https://mc.manuscriptcentral.com/e-videos .
